# Prognostic relevance of protein expression, clinical factors, and *MYD88* mutation in primary bone lymphoma

**DOI:** 10.18632/oncotarget.19936

**Published:** 2017-08-04

**Authors:** Yong Xu, Jian Li, Jian Ouyang, Juan Li, Jingyan Xu, Qiguo Zhang, Yonggong Yang, Min Zhou, Jing Wang, Cuiling Zhang, Yueyi Xu, Ping Li, Rongfu Zhou, Bing Chen

**Affiliations:** ^1^ Department of Hematology, Affiliated Drum Tower Hospital of Nanjing University Medical School, Nanjing, China; ^2^ Department of Hematology Oncology, Children’s Hospital of Nanjing Medical University, Nanjing, China

**Keywords:** primary bone lymphoma, JAK/STAT, MYD88

## Abstract

Primary bone lymphomas (PBLs) are composed of malignant lymphoid cells presenting in osseous sites, without supra-regional lymph node or extranodal involvement. We systematically characterized the immunophenotype and the myeloid differentiation factor 88 (*MYD88*)-L265P gene mutation status in PBL. Clinical data from 19 patients with PBL treated at Nanjing Drum Tower Hospital between 2009 and 2015 were analyzed retrospectively. Protein expression patterns were identified immunohistochemically, and *MYD88* mutation was assessed using polymerase chain reaction and direct DNA sequencing. Fifteen patients presented with diffuse large B-cell lymphoma. Clinical factors favoring a good prognosis were an age < 60 years and rituximab treatment. B-cell lymphoma 2 expression was detected in 5/15 diffuse large B-cell lymphoma patients, and was associated with a poor prognosis in a univariate model. Janus kinase/signal transducer and activator of transcription 3 (JAK/STAT3) signaling factors were upregulated in PBLs. All eighteen evaluable PBL samples harbored wild-type MYD88. These data thus suggest that age and rituximab treatment are independent prognostic factors determining overall survival, and that activation of JAK/STAT3 signaling may promote the pathogenesis of PBL. Moreover, the absence of *MYD88*-L265P mutation in PBL indicate there are distinct pathogenetic backgrounds among extranodal lymphomas.

## INTRODUCTION

Lymphoma initiating in the bone is rare, and is designated as primary bone lymphoma (PBL). PBL accounts for less than 7% of all malignant bone tumors, 5% of extranodal lymphomas, and 1% of all malignant lymphomas [l, 2]. According to the 2013 World Health Organization classification of bone/soft tissue tumors, a diagnosis of PBL signifies the presence of one or more skeletal tumors without visceral or regional lymph node involvement. The common sites of PBL are the femur, pelvis, humerus and tibia, but in rare cases, PBL can also affect the small bones of the limbs. Diffuse large B-cell lymphoma (DLBCL) is the most common histological subtype of PBL, while rare categories include follicular lymphoma, peripheral T-cell lymphoma, anaplastic large cell lymphoma (ALCL), extranodal natural killer/T cell lymphoma, and Burkitt’s lymphoma [1, 3–5]. Until now, only a few studies of PBL have included immunohistochemical and molecular investigations, and little is known about the prognostic significance of the *in situ* tissue antigen expression profiles in PBL.

In DLBCL, several well-characterized protein signaling cascades are of great benefit for targeted therapy strategies, and these signaling pathways can also serve as prognostic markers. For example, signal transducer and activator of transcription 3 (STAT3), a component of the Janus kinase and signal transducer and activator of transcription (JAK/STAT) signal-transducing pathway, is a downstream target of B-cell lymphoma 6 (BCL-6), which has been highly characterized in non-germinal center (GC)-derived DLBCL [6]. The overexpression of cellular myelocytomatosis oncogene (C-MYC; a downstream target of STAT3) integrated with the overexpression of B-cell lymphoma 2 (BCL-2) in DLBCL has been shown to influence the prognosis and predict the therapeutic response in patients receiving chemotherapy [7, 8]. Deregulation of the chemokine receptor 4 (CXCR4)/ proto-oncogene serine/threonine-protein kinases (PIM) cascade also seems to have prognostic and potential therapeutic value in DLBCL [9]. In addition, the activation of nuclear factor κB (NF-κB) occurs in most non-germinal center B-cell (non-GCB)-derived DLBCL cases [10], and the first NF-κB pathway inhibitors are currently being tested in clinical studies [11].

Through Sanger sequencing and whole-genome sequencing, a recurrent point mutation resulting in a single nucleotide substitution that changes a leucine to a proline at position 265 (L265P) in the myeloid differentiation factor 88 (*MYD88*) gene was detected in substantial percentage of DLBCLs, especially in extranodal DLBCLs [12–18]. This was determined to be a gain-of-function mutation that activates the NF-κB pathway to induce tumor cell survival. However, to the best of our knowledge, the frequency of *MYD88* mutations in PBL is still unknown because of the rarity of this tumor type.

In this study, we performed a mutation analysis of the *MYD88*-L265P mutation in 19 PBL patients, and investigated the immunophenotypic biomarkers and clinicopathological factors associated with PBL.

## RESULTS

### Clinicopathological features

Patient characteristics are shown in Table 1. The median age of the patients at diagnosis was 51 years (range, 15–78 years). The majority of them presented with DLBCL (*n* = 15; 78.9%), and these patients were further stratified into nine non-GCB patients and six GCB patients. In addition, two anaplastic lymphoma kinase (ALK)-positive ALCLs, one ALK-negative ALCL, and one mantle cell lymphoma (MCL) were identified. The most common presentation was bone pain (100%). Fourteen patients were classified as having unifocal bone disease, and the other five displayed multifocal bone involvement. The femur was the most commonly affected site in unifocal PBL patients (28.6%) at the time of presentation.

**Table 1:** Characteristics of the 19 PBL patients

Characteristics	*N*	%
**Age (years)**		
< 60	12	63.2
≥ 60	7	36.8
Sex		
Male	13	68.4
Female	6	31.6
**Histology**		
DLBCL	15	78.9
ALK-negative ALCL	1	5.3
ALK-positive ALCL	2	10.5
MCL	1	5.3
**Initial site (unifocal)**		
Femur	4	28.6
Pelvis	3	21.4
Spine	3	21.4
Other	4	28.6
Stage		
I/II	10	52.6
IV	9	47.4
**LDH**		
Normal	8	42.1
Elevated	11	57.9
**B symptoms**		
Yes	4	21.1
No	15	78.9
β2-MG		
Normal	13	68.4
Elevated	6	31.6
**IPI score**		
0–2	10	52.7
3–4	9	47.3

**DLBCL:** diffuse large B-cell lymphoma; **ALCL:** anaplastic large cell lymphoma; **ALK:** anaplastic lymphoma kinase; **MCL:** mantle cell lymphoma; **LDH:** lactate dehydrogenase; β2-MG: β2-microglobulin; **IPI:** International Prognostic Index

### Treatments and outcomes

Patient treatment regimens and outcomes are shown in Table 2. Seventeen patients received combined chemotherapy treatment, and two patients only received surgical treatment. Three patients received chemotherapy with radiotherapy. Three patients received chemotherapy as the initial treatment and cytokine-induced killer (CIK) treatment as consolidation therapy. Two patients (one stage-IVB DLBCL patient and one MCL patient) received chemotherapy as the initial treatment and autologous peripheral blood stem cell transplantation (auto-PBSCT) as consolidation therapy.

**Table 2:** Treatment regimens and OS/PFS of the 19 PBL patients

No.	Sex	Age	Treatment	Outcome	Survival time	
					**OS**	**PFS**
1	M	45	R-CHOP, CHOP	ANED	32	32
2	W	69	CHOP, R-CHOP, R-CHOP+MTX, GEMOX, CIK	ANED	11	5
3	M	69	CHOP	Died	15	5
4	M	30	CHOP, R-CHOP, ICE, RA	ANED	24	6
5	M	47	R-CHOP	ANED	60	60
6	W	69	B-CHOP, CHOP, CIK	ANED	54	54
7	M	32	CHOP	ANED	59	59
8	M	51	R-CHOP, Auto-PBSCT	ANED	15	15
9	W	53	SUR	Loss	2	2
10	W	68	R-CHOP, CIK	ANED	33	17
11	W	68	CHOP	Died	6	4
12	M	51	R-CHOP, R-DA-EPOCH, R-DHAP, GEMOX, RA	AWD	9	6
13	M	54	R-CHOP, ICE	ANED	24	24
14	M	45	R-CHOP, R-CHOP, R	ANED	26	26
15	M	23	CHOP	ANED	8	8
16	W	46	CHOP	ANED	48	48
17	M	78	CHOP, RA	AWD	7	4
18	M	15	SUR	ANED	54	54
19	M	51	R-CHOP, R-DHAP, Auto-PBSCT	ANED	10	10

**Remark:** RA: Radiation therapy; SUR: surgery; MTX: methotrexate;R-CHOP: Rituximab+CHOP; CIK: Cytokine-induced killer; Auto-PBSCT: Autologous peripheral blood stem cell transplantation; ICE: Ifosfamide+Cisplatin+Etoposide; GEMOX: Gemcitabine+Cisplatin; DHAP: cisplatin+Ara-C+dexamthsone;ANED: Alive with no evidence of disease; AWD: Alive with disease. OS: overall survival; PFS:progression free survival.

After the initial chemotherapy treatment, nine patients achieved complete remission (CR), seven patients achieved partial remission, and one patient experienced disease progression. By the last follow-up, two patients had died due to disease progression, while two patients were alive with the disease. Patients who received CIK treatment or auto-PBSCT were alive with no evidence of disease. One patient with ALK-positive ALCL had received surgical treatment alone, and he remained in CR for 54 months after surgery.

The median follow-up was 15 months, ranging from 2 months to 60 months. For all patients, the one-year overall survival (OS) and progression-free survival (PFS) rates were 94% and 67%, respectively, while the estimated two-year OS was 86%. For the 15 DLCBL patients, the one-year OS and PFS rates were 93% and 60%, respectively, and the estimated two-year OS was 83%. The results of univariate analysis of various factors influencing OS are shown in Table 3. Statistically significant factors that favorably influenced OS were an age < 60 years and treatment with rituximab.

**Table 3:** Univariate analysis of various factors affecting OS

Characteristics	OS (%)	*P* value
**Age (years)**		0.0069*
< 60 (*n* = 12)	100	
≥ 60 (*n =* 7)	71.4	
Sex		0.449
Male (*n =* 13)	92.3	
Female (*n =* 6)	83.3	
**LDH**		0.608
Normal (*n =* 9)	88.9	
Elevated (*n =* 10)	90.0	
**Stage**		0.781
I-II (*n =* 10)	90.0	
IV (*n =* 9)	88.9	
**DLBCL**		0.234
GCB (*n =* 6)	100	
Non-GCB (*n =* 9)	77.8	
**DLBCL treatment**		0.043*
Rituximab (*n =* 9)	100	
Without Rituximab (*n =* 6)	66.7	
**CR after initial treatment**		0.074
Yes (*n =* 10)	100	
No (*n =* 9)	77.8	

*Indicates statistical significance.

LDH: lactate dehydrogenase; DLBCL: diffuse large B-cell lymphoma; GCB: germinal center B-cell; CR: complete remission.

### Immunophenotypic studies and mutational status of *MYD88*-L265P

**Figure 1:**
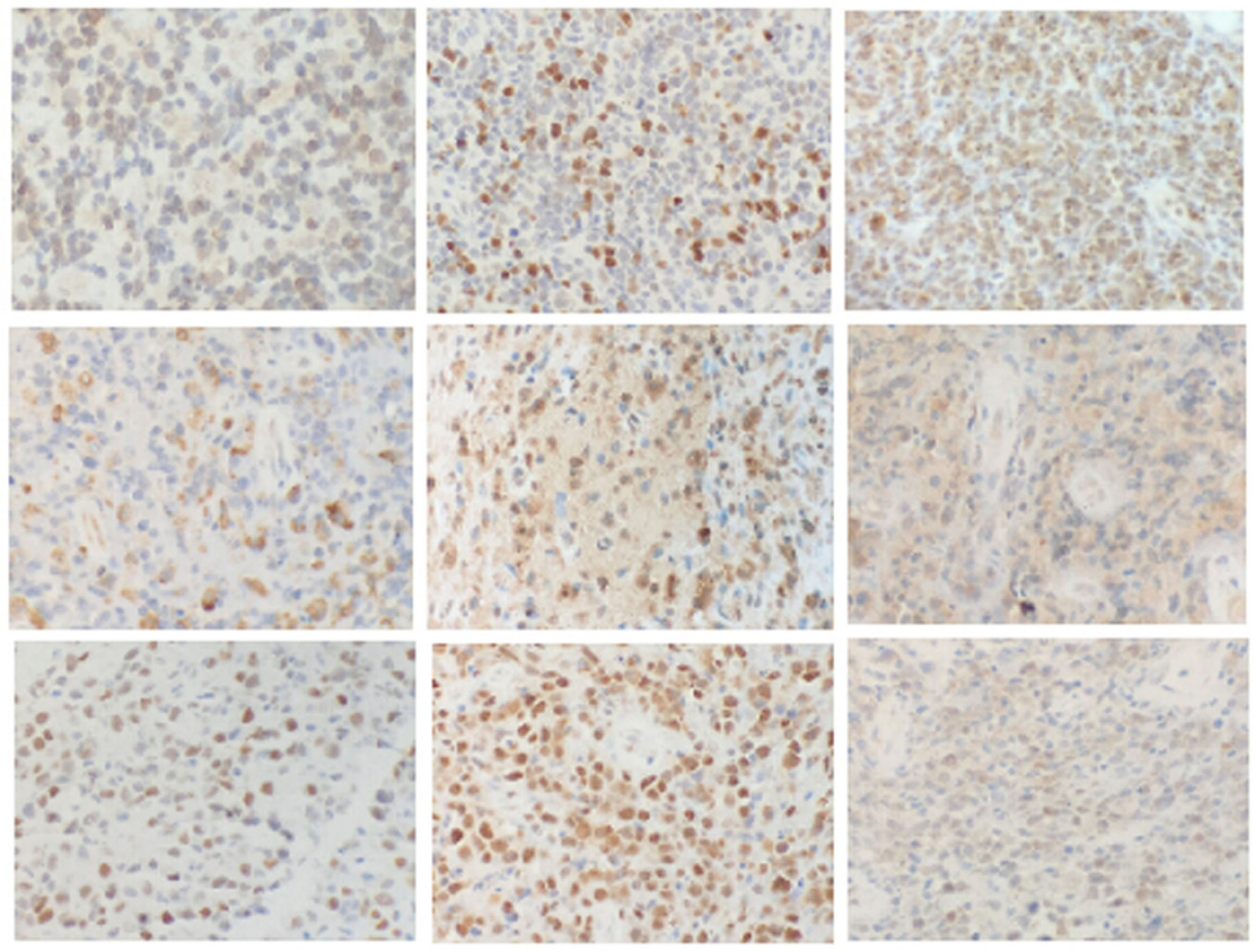
Immunohistochemistry of PBLs First row, left to right: BCL-2, C-MYC, and CXCR4. Second row, left to right: JAK2, P50, and P-P65. Third row, left to right: P53, PIM1, and PSTAT3. (200×).

The immunophenotypic characteristics of the 19 PBL cases are summarized in Table 4. Immunohistochemistry of a representative sample is presented in Figure 1. Cluster of differentiation 10 (CD10) and mutated melanoma-associated antigen 1 (MUM1) were positive in 4/15 (26.7%) and 8/15 (53.3%) DLBCLs, respectively, while BCL-2, BCL-6 and C-MYC were positive in 5/15 (33.3%), 10/15 (66.7%) and 7/14 (50%) DLBCLs, respectively. Seven of 18 cases expressed CXCR4, and 10/18 (55.6%) cases expressed PIM1. Proteins expressed in the canonical NF-κB pathway (P50 and P-P65) were also studied. P50 was expressed in 4/18 (22.2%) cases, and P-P65 was expressed in 6/18 (33.3%) cases. In the JAK/STAT pathway, PSTAT3 and JAK2 were evaluated. Nine of 18 evaluable cases expressed PSTAT3. JAK2, a kinase upstream of the STAT pathway, was highly positive in 10/18 (55.6%) cases.

**Table 4:** Biomarkers and MYD88-L265P mutation status of PBLs

Case	Histology	CD10	BCL-2	BCL-6	MUM-1	C-MYC	P53	P50	P65	PSTAT3	PIM1	CXCR4	JAK2	Subtype	MYD88	
															L265P AS-PCR	Sequence
1	**DLBCL**	−	+	+	+	+	**3+**	**2+**	**2+**	−	+	**2+**	+	NGCB	−	**L265P**
2	**DLBCL**	−	+	+	+	ND	ND	ND	ND	ND	ND	ND	ND	NGCB	−	**ND**
3	**DLBCL**	−	**3+**	+	+	**2+**	+	−	−	−	**3+**	−	+	NGCB	−	**L265P**
4	**DLBCL**	−	+	−	−	−	−	−	−	−	−	−	**2+**	NGCB	−	**L265P**
5	**DLBCL**	−	+	+	+	**2+**	−	−	−	−	**2+**	**2+**	+	NGCB	−	**L265P**
6	**DLBCL**	−	+	−	−	**2+**	−	+	**2+**	+	−	−	**3+**	GCB	−	**L265P**
7	**DLBCL**	−	+	−	+	**2+**	−	+	−	+	**3+**	**2+**	**2+**	NGCB	−	**L265P**
8	**DLBCL**	−	+	−	−	−	−	−	−	−	+	+	−	GCB	−	**L265P**
9	**DLBCL**	−	+	−	−	**3+**	−	−	−	−	+	−	−	NGCB	−	**L265P**
10	**DLBCL**	−	**2+**	+	+	**2+**	**2+**	**2+**	+	**2+**	**3+**	−	**2+**	NGCB	−	**L265P**
11	**DLBCL**	−	**3+**	+	+	**2+**	−	+	+	+	**3+**	−	**2+**	NGCB	−	**L265P**
12	**DLBCL**	2+	**3+**	**3+**	−	+	−	**3+**	**2+**	+	**2+**	**3+**	**2+**	GCB	−	**L265P**
13	**DLBCL**	+	**3+**	+	+	+	−	**2+**	**2+**	+	**2+**	**2+**	**3+**	GCB	−	**L265P**
14	**DLBCL**	+	+	+	−	−	−	−	−	−	−	−	+	GCB	−	**L265P**
15	**DLBCL**	+	+	+	−	+	**2+**	+	**3+**	**2+**	**3+**	**3+**	−	GCB	−	**L265P**
16	**ALK+ALCL**	ND	ND	ND	ND	+	−	−	−	−	**3+**	**2+**	−	−	−	**L265P**
17	**ALK-ALCL**	ND	ND	ND	ND	−	+	+	+	−	+	−	**3+**	−	−	**L265P**
18	**ALK+ALCL**	ND	ND	ND	ND	−	**2+**	+	+	**3+**	**3+**	−	**3+**	−	−	**L265P**
19	**MCL**	−	**3+**	**2+**	**2+**	**2+**	−	−	**3+**	+	+	−	**3+**	−	−	**L265P**

**Remark:** + = 5–25% positive tumor cells; 2+ = 25–75% positive tumor cells; 3+ = > 75% positive tumor cells.

*MYD88*: Myeloid differentiation factor 88; AS-PCR: Allele-specific polymerase chain reaction; DLBCL: Diffuse Large B-cell Lymphoma; GCB: germinal center B-cell; NGCB: non-germ inal center B-cell; ND: Not Done; ALK: Anaplastic Lymphoma Kinase; ALCL: Anaplastic Large Cell Lymphoma; MCL: mantle cell lymphoma.

**Figure 2:**
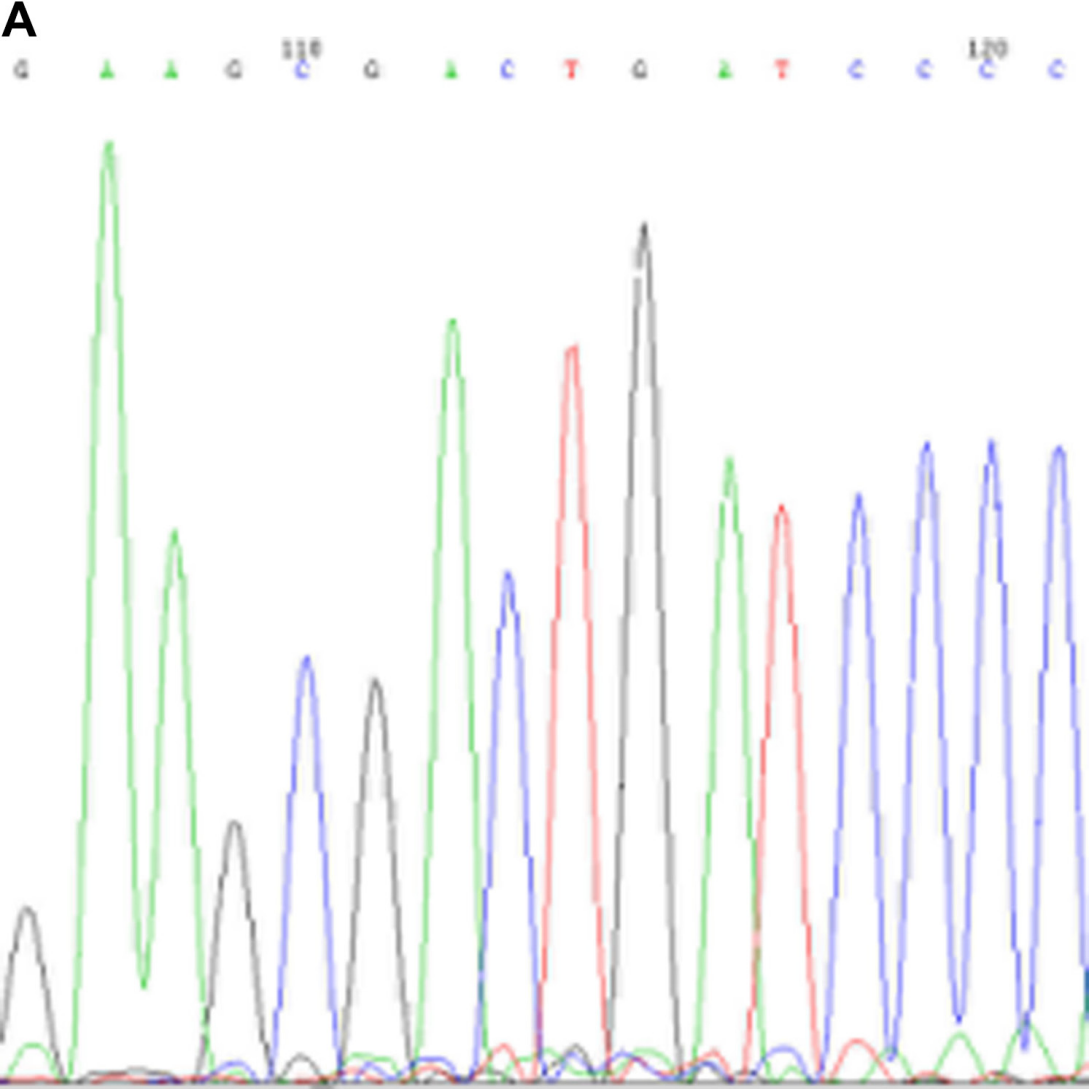

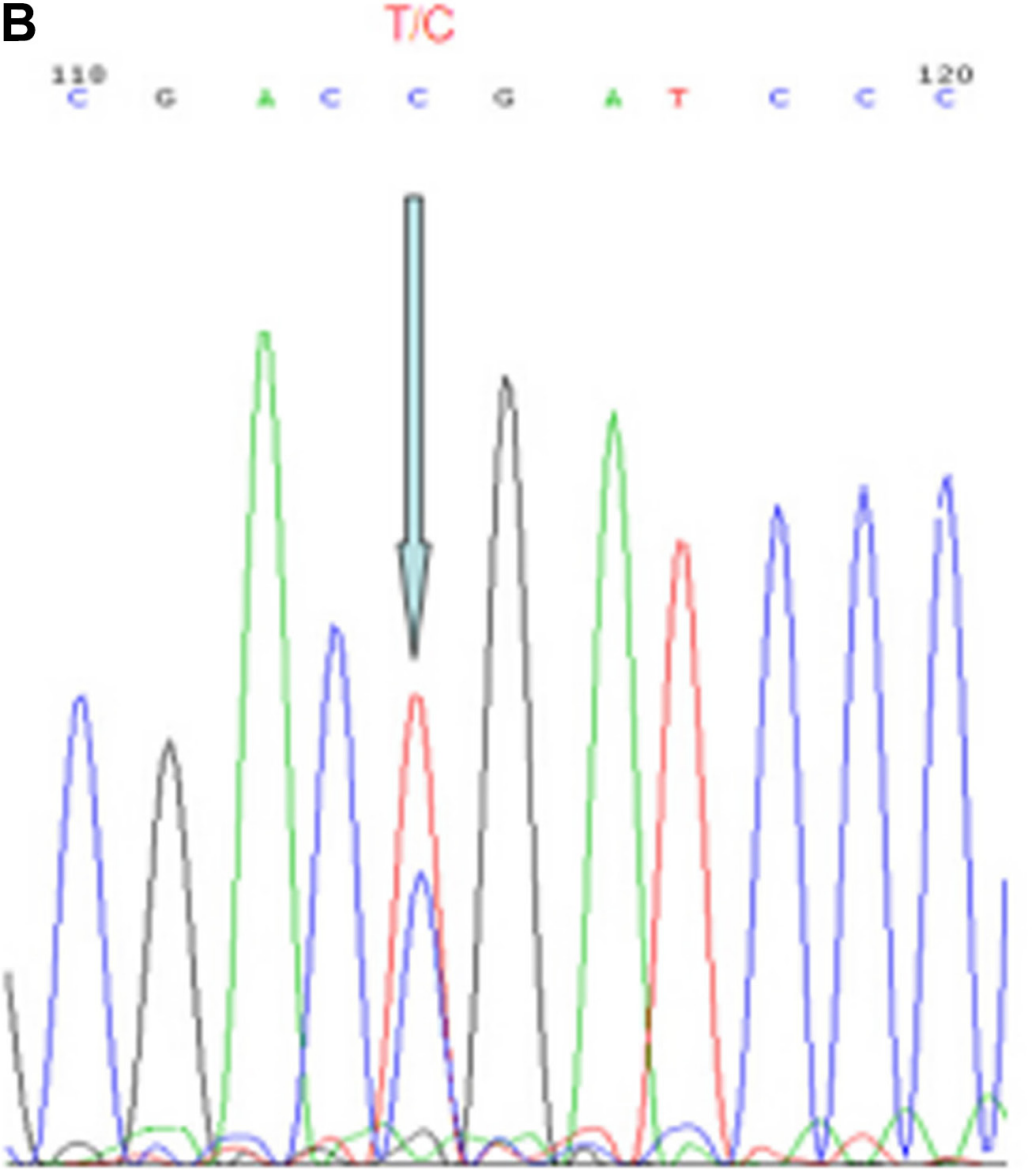
Representative results from Sanger sequencing for detection of the *MYD88*-L265P mutation Arrows indicate the wild-type genotype in PBL patients (**A**) and the heterozygous mutation in a testis DLBCL patient (**B**). *MYD88*: Myeloid differentiation factor 88.

*MYD88*-L265P mutational status is shown in Table 4, and a representative sequencing read is presented in Figure 2. Eighteen patients had sufficient DNA for *MYD88* mutation sequencing by a polymerase chain reaction and Sanger sequencing. All 18 PBL patients harbored wild-type *MYD88*.

### Biomarkers and clinicopathological correlations

The correlations between immunohistochemical markers/clinicopathological factors and OS are listed in Tables 5 and 3. The median follow-up period was 15 months. The majority of patients received cyclophosphamide, doxorubicin, vincristine, and prednisone (CHOP) alone (*n =* 6) or CHOP combined with rituximab (R-CHOP, *n =* 9) chemotherapy regimens. BCL-2 seemed to be a marker for poorer OS (*P* = 0.043). C-MYC expression, PIM1 expression and a complete response to initial treatment (*P* = 0.120, *P =* 0.085, *P =* 0.074, respectively) exhibited non-significant trends of correlation with OS. Other biomarkers investigated in this study did not correlate with survival. The factors that correlated with OS were chemotherapy combined with rituximab (*P* = 0.043) and an age < 60 years (*P* = 0.0069).

**Table 5:** Results of the biomarker statistical analyses (survival log-rank)

	Positive cases (%)	*P* value	Cut-off
CD10	4/15 (26.7)	0.470	+
MUM1	8/15 (53.3)	0.234	+
BCL-2	5/15 (33.3)	0.043*	++
BCL-6	10/15 (66.7)	0.220	+
C-MYC	7/14 (50)	0.120	++
P53	6/18 (33.3)	0.617	+
PSTAT3	9/18 (50)	0.906	+
P50	4/18 (22.2)	0.341	++
P65	6/18 (33.3)	0.362	++
PIM1	10/18 (55.6)	0.085	+++
CXCR4	7/18 (38.9)	0.245	++
JAK2	10/18 (55.6)	0.981	++

**Remark 1:** + = 5–25% positive tumor cells; ++ = 25–75% positive tumor cells; +++ = > 75% positive tumor cells. CD10, MUM1, BCL-2 and BCL-6 were only investigated in diffuse large B-cell lymphoma (DLBCL) patients.

**Remark 2:** CD10: cluster of differentiation 10; CD20: cluster of differentiation 20; MUM1: mutated melanoma-associated antigen 1; BCL-2: B-cell lymphoma 2; BCL-6: B-cell lymphoma 6; C-MYC: cellular myelocytomatosis oncogene; P53: phosphoprotein 53; PSTAT3: Phosphorylated signal transducer and activator of transcription 3; PIM1: proto-oncogene serine/threonine-protein kinases; CXCR4: chemokine receptor 4; JAK2: Janus kinase 2.

## DISCUSSION

PBL is a rare extranodal lymphoma subtype that was first described by Parker and Jackson as “reticulum cell sarcoma” [19]. With obvious improvements in imaging technology in recent decades, the proportion of patients diagnosed with PBL has increased. Here, we have described a series of 19 PBL patients from our medical institution. Patients may develop PBL at any age, but elderly patients seem to be affected more frequently. The male:female ratio has been nearly 1.5:1 in some of the most recent large studies, and a similar ratio was observed in our study [20–22]. The median age (51 years) of the patients in this study was similar to those in other clinical series [20–22]. In our series, 14 patients had unifocal bone disease, while the other 5 had multifocal bone involvement. The femur was the most commonly affected site in unifocal PBL patients (28.6%) at the time of presentation, followed by the pelvis (21.4%) and the spine (21.4%). No obvious clinical manifestations were found in early-stage PBLs; however, osteolytic lesions occurred more frequently in advanced disease.

PBL has been described as morphologically heterogeneous, and the overwhelming majority of cases have been characterized as B-cell lineage by immunohistochemistry. As in previous studies, DLBCL was the most common histological subtype (15/19) in our bone lymphoma series. Two extremely rare subtypes, MCL and ALK-negative ALCL, were also identified in our series.

PBL is a distinct subtype of non-hodgkin^,^s lymphoma and usually has favorable clinical features and a good prognosis. Several large studies have demonstrated that the OS of patients with PBL is significantly associated with disease stage and age [1, 3, 22]. In our series, OS did not differ significantly between stage I-II and stage IV patients, and this requires further investigation. Moreover, in the univariate analysis of prognostic factors in PBL, we found that an age ≥60 years was associated with shorter OS. In our study, the one-year OS and PFS rates of all patients were 94% and 67%, respectively, while the estimated two-year OS was 86% for all cases. These outcomes were comparable to those previously reported [1, 4, 5].

Different therapeutic schedules have been used to treat PBL, including surgery, chemotherapy, immunotherapy and radiotherapy. The role of surgery in PBL is limited to sample collection for histopathological diagnosis, stabilization and internal fixation of affected bones, and resolution of pathological fractures. Interestingly, one ALK+ALCL patient who only accepted surgical treatment was still alive without disease progression up to the last follow-up (54 months). Anthracycline-based chemotherapy such as CHOP (cyclophosphamide, doxorubicin, vincristine, prednisone), is the first-choice regimen for PBL-DLBCL. In our study, most of the DLBCL patients received CHOP or CHOP-like regimens and achieved a good CR rate. The survival benefit or potential complications of irradiating the affected bones after primary chemotherapy in patients with PBL are a matter of debate [5]. Prospective studies focused on this therapeutic issue in patients with PBL do not exist, thus, debate is mostly based on circumstantial evidence from large trials in nodal DLBCL. Here, a limitation of our study was that only three patients received radiation therapy after chemotherapy, due to disease progression.

Whether the anti-CD20 monoclonal antibody rituximab combined with a CHOP regimen can improve the prognosis of PBL has not been demonstrated. A few retrospective studies have suggested that the addition of rituximab is beneficial, as the three-year PFS rate was 50–60% after CHOP and 80–90% after R-CHOP [4, 23–24]. Other studies have reported no benefit from the addition of rituximab in patients with PBL [25]. In our study, patients who received rituximab had a better prognosis than those who did not. However, the effectiveness of rituximab in PBL therapy should be confirmed in further investigations, due to the long time-span and small sample size of our study.

Due to the good prognosis of PBL, hematopoietic stem cell transplantation is rarely applied in such patients. Herein, one MCL patient and one stage-IVB DLBCL patient who received up-front high-dose chemotherapy with auto-PBSCT achieved CR and had not relapsed by the last follow-up. Although only two patients received this treatment, in our opinion, it may be the treatment of choice for patients with poor prognostic factors.

A few series have studied the prognostic significance of the phenotypic and genetic characteristics of PBL [26, 27]. In these previous reports, approximately half of the PBL-DLBCL patients demonstrated a GCB phenotype by immunohistochemistry, with high BCL-2 and/or BCL-6 expression and relatively low MUM-1 expression. In our series, high percentages of BCL-2 and BCL-6 expression were also observed. Further analysis revealed that overexpression of BCL-2 was associated with a poor prognosis, consistent with previous reports. However, nearly half of the patients were positive for MUM1, which is higher than the incidence previously reported in the United States and Europe.

NF-κB is a transcription factor involved in several cellular survival pathways. It has been suggested that NF-κB activity contributes to the pathogenesis of different types of B-cell lymphoma, especially nodal non-GCB/activated-B-cell-type DLBCLs, although it is also encountered in a minority of GCB-type DLBCLs [28]. Recently, Koens et al. used immunohistochemical staining to study the NF-κB pathway in PBL [29]. They identified nuclear staining for P50 in 18% of PBL patients, but found no patients who were positive for C-REL or P65. The authors concluded that the NF-κB pathway is not an attractive therapeutic target in PBL. Similarly, in our study, P50 and P65 were strongly expressed in 22.2% and 33.3% of PBL patients, respectively, with no association with cellular origin. In further analysis, there was no correlation between OS and the expression of P50 or P65.

Previous reports have suggested that the overexpression of JAK2 is important in Hodgkin lymphoma and myeloproliferative neoplasms [30]. In our series, high expression of JAK2 was also observed in tumor cells, which may indicate that JAK2 is a prognostic biomarker in PBL. Furthermore, we discovered a high expression rate of C-MYC, PSTAT3, and PIM1 in PBL tumor cells. Although they had no significant correlation with OS, these proteins may also be prognostic markers, but this requires further investigation.

*MYD88* is an adaptor molecule for Toll-like receptor and interleukin-1 receptor signaling. Recently, next-generation DNA sequencing has demonstrated that functionally active mutations of *MYD88* are present in a substantial percentage of DLBCLs, and are more frequently detected in extranodal DLBCLs. The most common mutation is *MYD88*-L265P, which significantly contributes to the constitutive activation of the NF-κB pathway in lymphomagenesis [12–18]. Although the *MYD88* mutation has been investigated in several extranodal DLBCLs (such as primary central nervous system DLBCL, testicular DLBCL, cutaneous DLBCL (leg type), and breast DLBCL), studies on the *MYD88* mutation in PBL have been limited, and the clinical significance of the *MYD88* mutation has remained unclear. We are the first to report the *MYD88*-L265P mutation status of PBL patients. In our cohort, all 18 evaluable cases harbored wild-type *MYD88*, which implies that PBL has a relatively indolent clinical course and an excellent prognosis compared with other extranodal lymphomas. This further demonstrated that the NF-κB pathway may not be an attractive pathway for targeted therapy in PBL.

## MATERIALS AND METHODS

### Patients and tissue materials

Clinical histories and paraffin-embedded material from 19 patients presenting with PBL were retrieved from the archives and pathology department of the Nanjing Drum Tower Hospital. All cases were diagnosed and treated at the Nanjing Drum Tower Hospital between 2009 and 2015. Only cases fulfilling the criteria for the diagnosis of PBL (bone lesion ± local soft tissue component as the primary site of presentation, without evidence of disease elsewhere within a six-month period) were included in the study. Clinicopathological features including pathological patterns, sex, age and anatomic location were evaluated. The study was approved by the local ethics committee.

### Immunohistochemistry

The antibodies and conditions of the immunohistochemical staining are listed in Table 6. Immunohistochemical staining was performed on formalin-fixed, paraffin-embedded tissue (FFPE), and the EnVision System (DAKO, Glostrup, Denmark) was used for visualization. The relative proportion of positively-stained tumor cells was considered, but not the staining intensity. All markers were considered 1+ if 5–25% of the lymphoma cells were positive for expression, 2+ if 25–75% of the lymphoma cells were positive for expression, and 3+ if more than 75% of the lymphoma cells were positive for expression.

**Table 6:** Antibodies for immunohistochemistry

	Source	Dilution	Retrieval	Detection
CD10	Dako, Denmark	Prediluted	Citrate pH 6.0/pressure method	EnVision, Dako, Denmark
CD20	Dako, Denmark	Prediluted		
MUM1	Fuzhoumaixin, China	Prediluted		
BCL-2	Dako, Denmark	Prediluted		
BCL-6	Dako, Denmark	Prediluted		
C-MYC	Fuzhoumaixin, China	Prediluted		
P53	Fuzhoumaixin, China	Prediluted		
PSTAT3	Abcam, USA	1:150		
P50	Abcam, USA	1:300		
P65	Abcam, USA	1:500		
PIM1	Abcam, USA	1:300		
CXCR4	Abcam, USA	1:600		
JAK2	Abcam, USA	1:500		

**Remark:** CD10: cluster of differentiation 10; CD20: cluster of differentiation 20; MUM1: mutated melanoma-associated antigen 1; BCL-2: B-cell lymphoma 2; BCL-6: B-cell lymphoma 6; C-MYC: cellular myelocytomatosis oncogene; P53: phosphoprotein 53; PSTAT3: Phosphorylated signal transducer and activator of transcription 3; PIM1: proto-oncogene serine/threonine-protein kinases; CXCR4: chemokine receptor 4; JAK2: Janus kinase 2.

### DNA extraction

Five to ten 10-μm thick serial sections were cut from FFPE tissue blocks from 18/19 patients, and the most lymphoma cell-rich area was dissected with a scalpel. Total genomic DNA was subsequently extracted with the OMEGA FFPE DNA kit (Omega Bio-tek, Norcross, GA, USA) according to the manufacturer’s protocol.

### Mutational testing of *MYD88*-L265P

For *MYD88*-L265P mutation analysis, the following primers were used: forward primer, 5′-TTTGTGTGAGTGAATGTGTGC-3′; reverse primer, 5′-GCTGGCTCTGCTGGTCCTT-3′. The polymerase chain reaction conditions were 94°C for 10 min, followed by 10 cycles of 94°C for 50 s, 62°C for 50 s, and 72°C for 50 s, and 30 cycles of 94°C for 50 s, 56°C for 50 s, and 72°C for 50 s, with a final extension at 72°C for 10 min. DNA extracted from a testis lymphoma was used as a positive control. Sanger sequencing of the amplicon was performed with each forward primer, the BigDye Terminator v1.1 Cycle Sequencing Kit and the ABI3500DX Genetic Analyzer (Life Technologies, Indianapolis, IN, USA).

### Statistical analysis

Statistical analysis was performed with SPSS 19.0 software (Chicago, IL, USA). *χ*^2^ and Fisher’s exact tests were used to compare categorical variables between the two groups. The log-rank test was used to determine the prognostic impact of each immunophenotypic marker. Pearson’s chi-squared test and the Kaplan-Meier method were applied to determine the clinicopathological prognostic factors influencing OS. Results were considered to be statistically significant when *P* was < 0.05.
